# Retraction Note: Advancing breast cancer diagnosis with a near-infrared fluorescence imaging smart sensor for estrogen/progesterone receptor detection

**DOI:** 10.1038/s41598-026-49959-1

**Published:** 2026-04-28

**Authors:** Gong Zhang, Min Dong, Xiulei Yao, Yuke Xia, Han Yu, Yu zhou, Chao Lian, Yunlei Zhang, Yiyao Cui

**Affiliations:** 1 Department of Thyroid and Breast Surgery, Department of Ultrasound, Central Laboratory, Translational Medicine Research Center, The Affiliated JiangNing Hospital of NanJing Medical University, Nanjing, 211100 China; 2 Department of Comparative Medicine, Jinling Hospital, School of Medicine, Nanjing University, Nanjing, 210002 China; 3 The Key Laboratory of Clinical and Medical Engineering, School of Biomedical Engineering and Informatics, Nanjing Medical University, Nanjing, 211100 China

Retraction of: *Scientific Reports* 10.1038/s41598-023-48556-w, published online 30 November 2023

The Editors have retracted this Article. After publication, concerns were raised regarding the veracity of data presented in this work. Specifically, portions of the middle and bottom immunohistochemistry panels in Figure 5C appear to overlap. Furthermore, the ethics approval documentation provided by the Authors is not consistent with the ethics statements in the Article’s Methods. The Editors therefore no longer has confidence in the results and conclusions of this Article.

Gong Zhang, Yunlei Zhang, and Yiyao Cui do not agree with this retraction. None of the remaining Authors have responded to correspondence from the Editors about the retraction.

